# Spontaneous coronary artery dissection in a male patient with haemophilia A: a case report

**DOI:** 10.1093/ehjcr/ytaf196

**Published:** 2025-04-16

**Authors:** Mitsukuni Kimura, Yasuhiro Nakano, Kotaro Abe, Tetsuya Matoba

**Affiliations:** Department of Cardiovascular Medicine, Faculty of Medical Sciences, Kyushu University, 3-1-1 Maidashi, Higashi-ku, Fukuoka 812-8582, Japan; Department of Cardiovascular Medicine, Faculty of Medical Sciences, Kyushu University, 3-1-1 Maidashi, Higashi-ku, Fukuoka 812-8582, Japan; Department of Cardiovascular Medicine, Faculty of Medical Sciences, Kyushu University, 3-1-1 Maidashi, Higashi-ku, Fukuoka 812-8582, Japan; Department of Cardiovascular Medicine, Faculty of Medical Sciences, Kyushu University, 3-1-1 Maidashi, Higashi-ku, Fukuoka 812-8582, Japan

**Keywords:** Spontaneous coronary artery dissection, Haemophilia, Coronary computed tomographic angiography, Case report

## Abstract

**Background:**

Spontaneous coronary artery dissection (SCAD) is characterized by the unexpected formation of an intramural haemorrhage within the wall of an epicardial coronary artery with or without an intimal tear, resulting in an acute coronary syndrome. Haemophilia A and B are inherited X-linked recessive bleeding disorders caused by the absence or dysfunction of clotting factors VIII (FVIII) or IX.

**Case summary:**

A 50-year-old male patient with a history of haemophilia A, type 2 diabetes and smoking presented to our facility with prolonged chest pain. His laboratory results revealed increased myocardial biomarkers and an elevated activated partial thromboplastin time despite the absence of previous bleeding tendencies or coagulation factor replacement therapy. Non-ST-elevation myocardial infarction was suspected, and coronary angiography detected severe stenosis in the left circumflex artery. Intravascular ultrasound confirmed an intramural haematoma without an intimal tear, resulting in an SCAD diagnosis. The patient, with no bleeding complications, was conservatively treated with medications, including aspirin and bisoprolol. A coronary computed tomographic angiography (CCTA) on day 3 revealed haematoma resolution, and the patient was discharged on day 11. CCTA at 8 months detected the disappearance of the previously seen intramural haematoma.

**Discussion:**

We revealed two important clinical issues: (i) SCAD in a male patient with haemophilia and (ii) the usefulness of CCTA in observing SCAD lesions.

**Learning points**

- Haemophilia is more common in males, but SCAD, which occurs more frequently in females, can also develop.
- In haemophilia patients, there are several concerns during coronary angiography, such as the need for clotting factor replacement and the risk of haemorrhagic complications, but CT may be useful for evaluating dissection lesions.

## Introduction

Spontaneous coronary artery dissection (SCAD) is characterized by the unexpected formation of intramural haemorrhage within the wall of an epicardial coronary artery with or without an intimal tear, resulting in an acute coronary syndrome (ACS).^1^ Approximately 90% of SCAD cases occur in women, and SCAD is a frequent cause of acute myocardial infarction (AMI) in young women.^1^ A recent study hypothesized that the primary mechanism of SCAD is a disruption of the vasa vasorum resulting in the secondary intramural haemorrhage to form a haematoma, which may obstruct the bloodstream.^2^ Haemophilia A and B are inherited X-linked recessive bleeding disorders caused by the absence or dysfunction of clotting factors VIII (FVIII) or IX. Some cohort studies revealed that patients with haemophilia are more likely to develop coronary artery disease, especially AMI, at a younger age despite the presence of bleeding disorders.^3,4^ However, the mechanisms by which patients with haemophilia suffer from the early onset of cardiovascular disease remain unknown.

Here, we report the first case of a patient with haemophilia who suffered AMI due to SCAD. We paid special attention to antithrombotic therapy during catheterisation. Further, we used high-resolution coronary computed tomographic angiography (CCTA) to observe the SCAD lesion during the acute and healing phases, which seemed useful for SCAD lesion observation, especially in cases with a high risk of cardiac catheterisation.

## Summary figure

**Figure:**
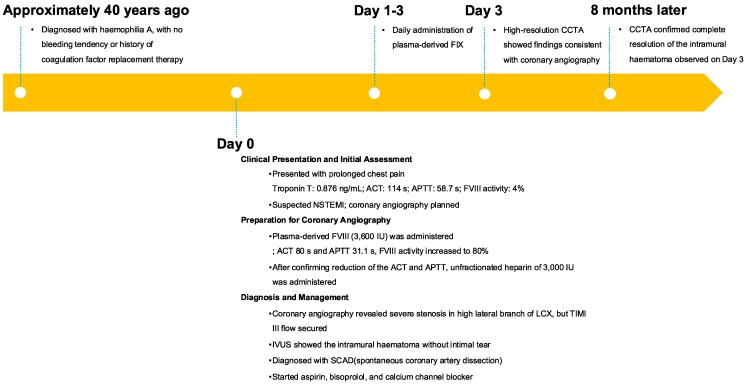


## Case presentation

**Figure 1:**
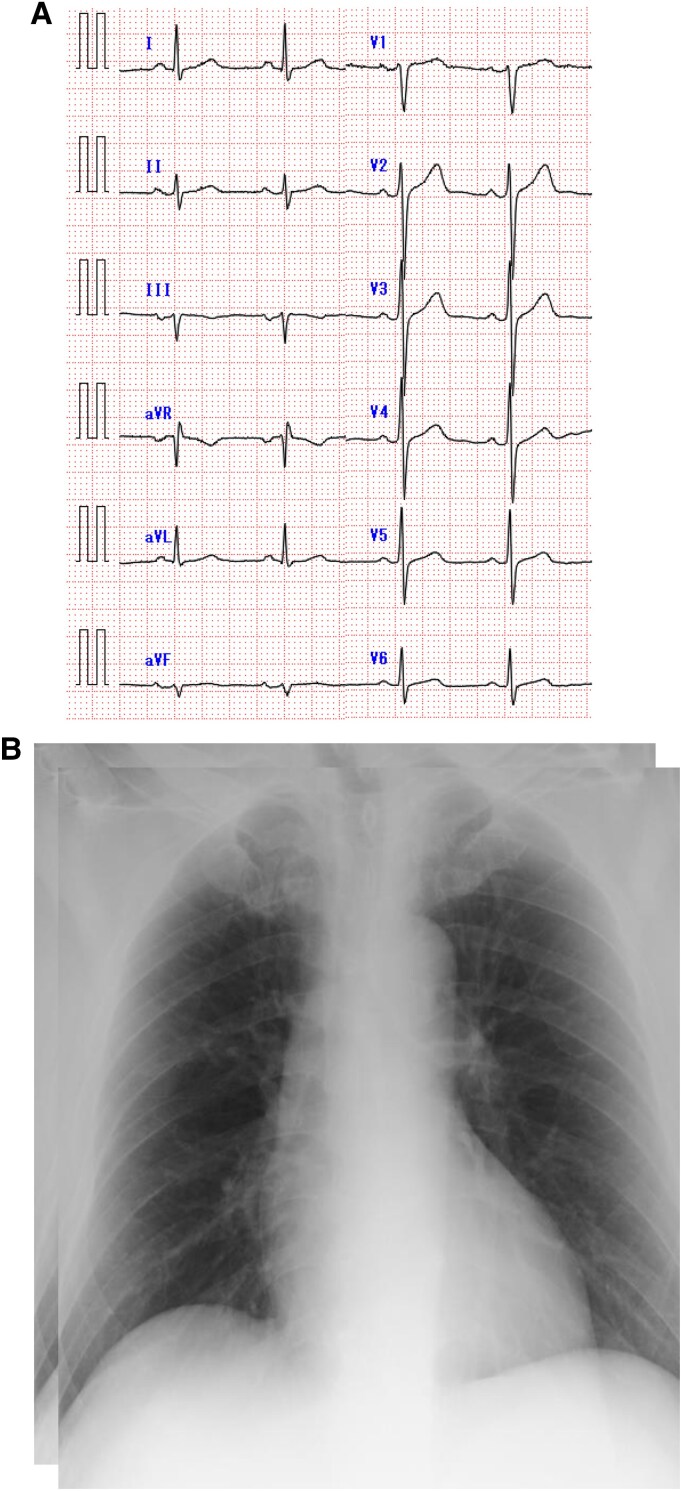
(*A*) Electrocardiogram upon admission. (*B*) Chest X-ray upon admission.

**Figure 2:**
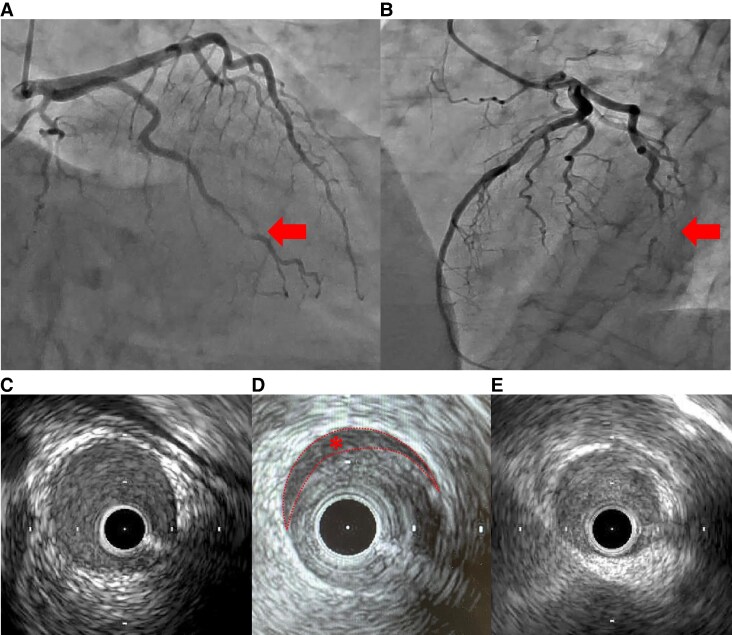
Angiography and IVUS of the left coronary artery. (*A*) and (*B*) Angiography demonstrating severe stenosis of the high lateral branch of the left circumflex artery. (*C*) The IVUS image of the proximal segment, (*D*) the culprit lesion and (*E*) the distal segment. IVUS image of the culprit lesion exhibited a false lumen (*).

**Figure 3:**
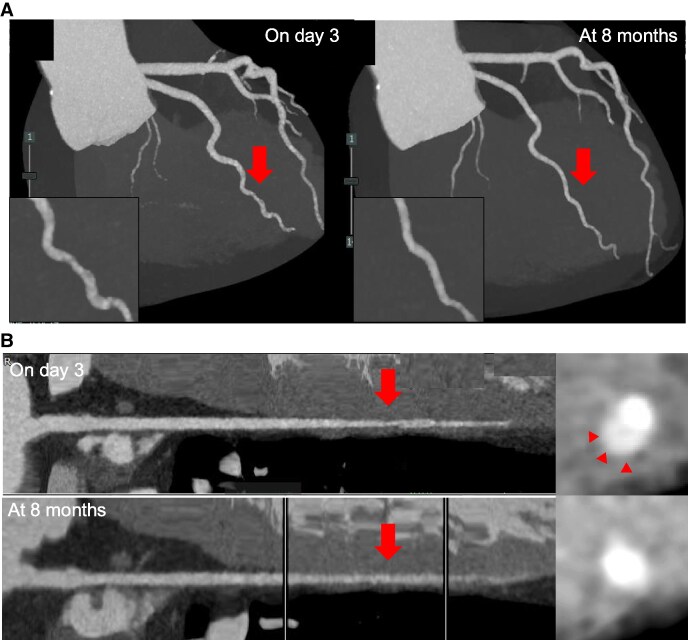
(*A*) Maximum intensity projection (MIP) image of CCTA. Left: MIP image demonstrating dissection flaps and stenosis on day 3. Right: a healed lesion at 8 months (arrows). (*B*) Curved planar reformation (CPR) image of the CCTA. Upper panel: CPR image showing intramural haematoma in the culprit lesion on day 3 (arrows). Lower panel: Absence of haematoma at 8 months.

A 50-year-old male patient visited our hospital because of sudden prolonged chest pain. He was diagnosed with haemophilia A in childhood, but demonstrated no bleeding tendency and was not treated with coagulation factor replacement therapy. He was a current smoker, reporting a history of type 2 diabetes. His blood pressure was 132/84 mmHg, and his pulse rate was 73 bpm. Electrocardiography demonstrated no significant ST-segment elevation or depression (*Figure 1A*). Laboratory examination detected increased myocardial biomarkers, including serum troponin T at 0.876 ng/mL (normal < 0.014), creatine kinase at 601 U/L (normal >59 to < 248), and creatine kinase-MB at 77 U/L (normal < 12). The prothrombin time and international normalized ratio were normal. However, the APTT was increased at 58.7 s (normal < 35), and the factor VIII activity was 4% (normal >60 to <150) (*Table 1*). The chest X-ray revealed no cardiac enlargement or pulmonary congestion (*Figure 1B*). Echocardiography demonstrated no local asynergy and almost normal left ventricular systolic function with a left ventricular ejection fraction of 60%. Hence, NSTEMI was suspected. The patient had persistent chest symptoms after the arrival at the hospital; thus, we decided to perform coronary angiography using a right radial approach after administering plasma-derived VIII (rurioctocog alfa, Takeda Pharmaceuticals) of 3600 IU (40 IU/kg), resulting in an VIII activity of 80%. The ACT was reduced from 114 to 80 s and APTT was reduced from 58.7 to 31.1 s after 15 min of administration (*Table 1*). Unfractionated heparin at 3000 IU was administered after local anaesthesia with lidocaine and a 6F introducer sheath insertion. Coronary angiography revealed single-vessel disease with a severe stenosis of the high lateral branch of the left circumflex artery (*Figure 2A, B*). The stenosis remained unchanged despite the intracoronary administration of nitro-glycerine, and atherosclerosis was mild. Hence, we proceeded with intracoronary imaging using Intravascular ultrasound (IVUS) (AnteOwl, Terumo Corp). This imaging detected the presence of an intramural haematoma and the absence of an intimal tear (*Figure 2C, D, E*). No evidence indicated plaque rupture or erosion, which could have caused the ACS. Therefore, we diagnosed this patient with NSTEMI related to SCAD. Thrombolysis in myocardial infarction III flow was secured; thus, we adopted a conservative approach. We started aspirin (ASA) and bisoprolol for this patient to treat SCAD as well as a calcium channel antagonist to control blood pressure after coronary angiography. The peak serum creatine kinase level was 601 mg/dL. We administered plasma-derived FXIII daily until post-operative day 3, but FXIII administration was completed without any bleeding complications after ASA administration; thus, we decided not to administer a coagulation factor replacement therapy. An ultra-high-resolution CCTA^5^ was performed on day 3 (Aquilion Precision, Canon, Inc.), and this CCTA revealed similar findings to coronary artery imaging. The patient underwent abdominal echography as part of the screening for extracoronary arthropathies (especially fibromuscular dysplasia), which revealed no abnormal results. He was discharged on day 11. CCTA at the 8-month follow-up detected the disappearance of the intramural haematoma seen on day 3, as well as lesion resolution (*Figure 3A, B*).

**Table 1:** Laboratory test results

	Day 0	Day 0	Day 1	Day 3	Normal values
	The arrival at hospital	15 min after administration of plasma-derived FXIII			
CK (U/L)	601	508	393	191	59–248
CK-MB (U/L)	77	67	49	13	<12
Troponin T (ng/mL)	0.876	0.810	0.740	0.543	<0.014
PT-INR	0.89	1.04	1.02	1.08	0.80–1.20
APTT (second)	58.7	31.1	34.2	39.3	<35.0
Factor VIII activity (%)	4	—	53	—	60–150

Abbreviations: APTT, activated partial thromboplastin time; CK, creatine kinase; PT-INR, prothrombin time and international normalized ratio.

## Discussion

We revealed two important clinical issues: (i) SCAD in a male patient with haemophilia and (ii) the usefulness of CCTA to observe SCAD lesions.

First, patients with haemophilia can develop MI due to SCAD. The cause of SCAD frequently includes pre-disposing factors associated with vascular vulnerabilities (e.g. fibromuscular dysplasia, connective tissue disorder or inherited tissue disorder) and precipitating factors such as emotional stress, physical stress (e.g. from an extreme Valsalva manoeuvre, retching, vomiting, coughing or isometric exercise), the use of stimulant medications or illicit drugs and hormonal triggers (e.g. pregnancy).^6^ However, 90% of SCAD cases occur in women; thus, the pre-disposing factors of male SCAD cases remain unclear. The proportion of fibromuscular dysplasia is generally lower and physical exertion may trigger SCAD in male patients.^7^ In this case, IVUS findings revealed an intramural haematoma without an intimal tear despite the absence of obvious pre-disposing or precipitating factors except for hypertension, which is predominantly observed in patients with haemophilia. Further, the lesion was flanked by arteries of normal calibre, resulting in a type 2A SCAD diagnosis.^1^ Considering the hypothesis that the primary mechanism of SCAD is a vasa vasorum disruption resulting in secondary intramural haemorrhage forming a haematoma, in patients with haemophilia, the pre-disposition to bleeding may contribute to the expansion of the haematoma, potentially causing SCAD to develop AMI. Previous cohorts reporting that patients with haemophilia developed MI at a young age did not include coronary imaging data, and these reports were conducted when SCAD was not well recognized. Therefore, young patients with haemophilia who developed MI may include SCAD cases. The present case does not provide a causal association between SCAD and haemophilia, but reports indicated spontaneous dissections in cervical vessels in a patient with haemophilia.^8^ Additional research is required to confirm any potential connection between SCAD and haemophilia, but this report is important to recognize that young patients with haemophilia may be at risk of MI due to SCAD.

Second, CCTA may be a useful means of observing dissection lesions in patients with haemophilia having SCAD. In patients with haemophilia undergoing catheterisation, pre- and post-procedural coagulation factor supplementation is required, and careful attention must be paid to haemorrhagic complications such as haematoma formation at the puncture site. In our case, ultra-high-resolution CCTA confirmed both the flap in the acute phase and its resolution in the chronic phase. Catheterisation is undoubtedly the gold standard for diagnosing SCAD, but CT may be effectively used for follow-up.

## Conclusions

We reported the first case of a patient with haemophilia who developed AMI due to SCAD, indicating an interaction in the pathogenesis. A high-resolution CCTA was useful to follow-up on the SCAD lesion.

## Supplementary Material

ytaf196_Supplementary_Data

## Data Availability

The data underlying this article are available in the article and its online Supplementary material.

## Lead author biography

Mitsukuni Kimura was born on 6 July 1992. In 2017, he graduated from Kyushu University. From 2017 to 2019, he became a junior and senior resident in Saiseikai Fukuoka GeneralHospital. From 2020 to 2021, he works at Oita prefecture hospital as senior resident. From 2021, he conducted research coronary calcification at Kyushu University Graduate School of Medical Sciences.

## Supplementary material

**Consent:** The patient signed written informed consent for the publication of this report and accompanying images in line with the COPE guidelines.

**Funding:** This work was supported by the Japan Society for the Promotion of Science (JSPS) KAKENHI grant number JP 23754577 (23K08454) to T.M.

## References

[1] Kim ESH . Spontaneous coronary-artery dissection. N Engl J Med 2020;383:2358–2370. 33296561 10.1056/NEJMra2001524

[2] Jackson R, Al-Hussaini A, Joseph S, Van Soest G, Wood A, Macaya F. Spontaneous coronary artery dissection: pathophysiological insights from optical coherence tomography. JACC Cardiovasc Imaging 2019;12:2475–2488. 30878439 10.1016/j.jcmg.2019.01.015

[3] Pocoski J, Ma A, Kessler CM, Boklage S, Humphries TJ. Cardiovascular comorbidities are increased in US patients with haemophilia A: a retrospective database analysis. Haemophilia 2014;20:472–478. 24286307 10.1111/hae.12339

[4] Girolami A, Ruzzon E, Fabris F, Varvarikis C, Sartori R, Girolami B. Myocardial infarction and other arterial occlusions in patients with haemophilia A: a cardiological evaluation of all 42 cases reported in the literature. Acta Haematol 2006;116:120–125. 16914907 10.1159/000093642

[5] Oostveen LJ, Boedeker KL, Brink M, Prokop M, de Lange F, Sechopoulos I. Physical evaluation of an ultra-high-resolution CT scanner. Eur Radiol 2020;30:2552–2560. 32040726 10.1007/s00330-019-06635-5 PMC7160079

[6] Hayes SN, Tweet MS, Adlam D, Kim ESH, Gulati R, Price JE, et al Spontaneous coronary artery dissection: JACC state-of-the-art review. JACC 2020;76:961–984. 32819471 10.1016/j.jacc.2020.05.084

[7] Khalili H, Hanzel GS. Periprocedural bleeding in patients undergoing WATCHMAN device placement. JACC Cardiovasc Interv 2016;9:865–866. 27101916 10.1016/j.jcin.2016.02.023

[8] Iacono S, Baschi R, Di Giorgi L, Gagliardo C, Pezzini A, Monastero R. Internal carotid artery dissection in a patient with haemophilia A: a case report and literature review. Neurol Sci 2023;44:1765–1768. 36795298 10.1007/s10072-023-06671-6

